# Rethinking HIV-prevention for school-going young people based on current behaviour patterns

**DOI:** 10.1080/17290376.2017.1376704

**Published:** 2017-09-21

**Authors:** Maretha Visser

**Affiliations:** ^a^ PhD, is a professor in Psychology at the Department of Psychology, University of Pretoria, Pretoria, South Africa

**Keywords:** HIV-prevention, sexual risk behaviour, school going youth, perceived social norms, community gender norms, South Africa, Prévention du VIH, comportement sexuel à risque, jeunesse scolaire; perception des normes sociales, normes communautaires à l’égard des genres, Afrique du Sud

## Abstract

The aim of the research was to gain increased knowledge regarding the sexual risk behaviour of school-going young people in South Africa after two decades of HIV-education in schools, to contribute to the development of improved HIV prevention strategies. In collaboration with the Department of Education, a sample of 5305 learners (between 10 and 18 years in Grades 5–12) from high-risk communities were identified. They completed a survey that assessed self-reported sexual risk behaviour and variables that potentially underlie sexual risk, such as attitudes towards preventive behaviour, perceived social norms and self-efficacy (based on the theory of planned behaviour [TPB]) and social factors like caregiver relationships and gender norms (based on the social ecological theory). Lifetime sex was reported by 49.4% of boys and 30.5% of girls in Grades 8–12, while 56% of the sexually active young people reported consistent condom use. Accurate knowledge about HIV transmission was low (37.8%). Regression analysis showed that risk behaviour was more prominent among older male youths, who perceived social norms as encouraging sexual activity, who use alcohol excessively, and who have negative attitudes towards abstinence. Perceived traditional community gender norms and negative relationships with caregivers were also associated with sexual risk behaviour. This research showed that the TPB can be used in planning HIV prevention interventions for young people. It also revealed that HIV-prevention strategies should focus beyond educating the individual, to address community factors such as improving caregiver relationships, the culture of substance abuse, peer group norms and inequality in community gender norms. These community processes influence young people's behaviour and need to be addressed to allow the youth to make healthy behavioural choices.

## Résumé

L'objectif de la recherche est d'acquérir une connaissance accrue des comportements sexuels à risque des jeunes scolarisés en Afrique du Sud après deux décennies d'éducation sur le VIH offerte à l’école, afin de contribuer au développement de stratégies visant à améliorer la prévention du VIH. En collaboration avec le ministère de l'Éducation, un échantillon de 5305 apprenants (entre 10 et 18 ans et qui étaient entre la 5e et la 12e année de leur cursus scolaire) provenant de communautés à haut risque ont été identifiés. Ils ont complété un questionnaire qui a évalué les comportements sexuels à risque auto-déclarés ainsi que les facteurs qui sont en rapport avec les risques sexuels, tels que les attitudes à l'égard des comportements préventifs, la perception des normes sociales et l'auto-efficacité (selon la théorie du comportement planifié) ainsi que des facteurs sociaux comme les relations avec les travailleurs sociaux et les normes liées au genre (basées sur la théorie de l'écologie sociale). Le sexe à vie a été signalé par 49,4% des garçons et 30,5% des filles de la 8e à la 12e année, tandis que 56% des jeunes sexuellement actifs ont déclaré qu’ils utilisaient de manière constante le préservatif. Une connaissance précise de la transmission du VIH était faible (37,8%). L'analyse de régression a montré que les comportements à risque étaient plus importants parmi les jeunes hommes âgés, qui sont d’avis que les normes sociales encouragent une activité sexuelle ; par ailleurs, ils consomment l'alcool de manière excessive et ont des attitudes négatives à l'égard de l'abstinence. La perception des normes traditionnelles de la communauté à l’égard du genre et les relations négatives avec les travailleurs sociaux étaient également associées à un comportement sexuel à risque. Cette recherche a montré que la théorie du comportement planifié peut être utilisée dans la planification des interventions de prévention du VIH pour les jeunes. Il a également révélé que les stratégies de prévention du VIH devraient aller au-delà de l'éducation de l'individu, pour aborder les facteurs communautaires tels que l'amélioration des relations avec les travailleurs sociaux, la culture de la toxicomanie, les normes du groupe de pairs et l'inégalité dans les normes communautaires à l’égard du genre. Ces processus communautaires influent sur le comportement des jeunes et doivent être pris en compte pour permettre aux jeunes de faire des choix de comportement sains.

## 1. Introduction

South Africa has one of the highest HIV prevalence rates in the world. It is estimated that about 6.8 million people in South Africa (18.9% of the population between 15 and 49 years) are infected (UNAIDS, 2014a). The most recent national household survey (Shisana et al., 2014) estimated that 7.1% of young people from 15 to 24 years are infected. Although the disease has stabilised over the past five years, 1.7% of the population gets newly infected each year (Shisana et al., 2014). A large proportion of new infections is among young people (15–24 years) (United Nations Children’s Fund, 2013), especially young women (24.1%) (Shisana et al., 2014; UNAIDS, 2014b). AIDS is currently the leading cause of death among adolescents in Africa (Bekker, Johnson, Wallance, & Hosek, 2015). Young people are at the centre of the global HIV and AIDS epidemic, especially regarding opportunities for HIV prevention (Bekker et al., 2015; Manosch & Mahy, 2006).

HIV education has been formally implemented in schools in the past two decades (Department of Basic Education, 1998, 2003, 2011). The interventions varied from short-term awareness and information campaigns to large-scale implementation of life skills and peer education programmes and the institutionalisation of the subject Life Orientation as part of the school curriculum. The persistently high levels of new infections among young people show that these interventions are not effective in addressing HIV risk. They are not addressing the underlying reasons for new infections successfully (Stirling, Rees, Kasedde, & Hankins, 2008). For school-based HIV-prevention strategies to be effective among young people, it should be based on an understanding of young people's sexual risk behaviour and the underlying drivers for these behaviours. This research, done in collaboration with the Department of Basic Education, aims to understand young people's sexual risk behaviour and the underlying factors influencing their actions.

Several South African studies highlighted the discrepancy between HIV awareness and reported sexual risk behaviour of young people (James, Reddy, Taylor, & Jinabhai, 2004). The Youth Risk Behaviour Survey (YRBS) (Reddy et al., 2013) reported that 36% of learners in Grades 8–11 reported being sexually active and only 33% of them reported consistent condom use. High levels of early sexual debut (before 15 years of age) was consistently reported over the past decade (Pettifor, O’Brien, MacPhail, Miller, & Rees, 2009; Reddy et al., 2013; Shisana et al., 2014). Twelve per cent learners reported early sexual debut (Reddy et al., 2013). Shisana et al. (2014) highlighted intergenerational sex (partner five years older) reported by 33.7% of young females (15–19 years) and multiple sexual partners reported by 37.5% of young men (15–24 years) as contributing to young people's HIV risk. Increasing condom use at last sexual encounter was regarded as a success story in HIV prevention among young people (Shisana et al., 2009), but condom use was found to decline again during the past five years to 67.5% of males and 49.8% of females (15–24 years) reporting condom use at last sex (Shisana et al., 2014).

Numerous studies have been conducted to understand young people's sexual risk behaviour. Many interacting individual, relational, and environmental factors were identified that influence high-risk sexual behaviour (Eaton, Flisher, & Aaro, 2003; Harrison, Cleland, Gouws, & Frohlich, 2005; Selikow, Ahmed, Flisher, Mathews, & Mukoma, 2009):

1. Factors within the person (psychological factors such as cognitions, self-efficacy, self-esteem, low-risk perception).
2. Relational factors (such as interpersonal environment, peer pressure, lack of role models).
3. Social and structural factors (gender norms, shared beliefs and values, legal, political, economic, and organisational elements of society).

Peer pressure among young people unmistakably undermines healthy social norms and HIV prevention messages (Selikow et al., 2009). Gender inequalities and social norms about sexuality (Jewkes, Dunkle, Nduna, & Shai, 2010; Mantell et al., 2006; Strebel et al., 2006), sexual coercion, alcohol use (Harrison, Newell, Imrie, & Hoddinott, 2010), and economic conditions (Dinkelman, Lam, & Leibbrandt, 2008; Ofosu-Amaah, Egamberdi, & Dhar, 2009) were prominent factors underlying HIV risk behaviour.

Despite large volumes of research on HIV among young people in Southern Africa, there is no evidence-based best practice model for HIV-prevention among the school-going population yet (Harrison et al., 2010). One indisputable guideline for HIV-prevention is that programmes should be grounded in the understanding of adolescent sexual behaviour and empirical data that may explain adolescent risk-taking behaviour. Prevention strategies should be targeted at specific risk factors underlying behaviour in the context (Gupta, Parkhurst, Ogden, Aggleton, & Mahal, 2008; Rotheram-Borus, Swendeman, & Chovnick, 2009; Taylor, 2004). Gupta et al. (2008, p. 773) stressed that ‘context really does matter and that a combination of successful approaches in one place might not be transferable to another’. This research was conducted to investigate the underlying reasons for school-going young people's sexual risk behaviour to inform a new generation of HIV-prevention strategies.

The social-cognitive theory of planned behaviour (TPB) (Ajzen, 1991) and social ecological theory (SET) (Bronfenbrenner, 1995) was used to understand the various factors underlying sexual risk behaviour of young people. According to the TPB, behaviour is influenced by an individual's behavioural intentions, which is in turn influenced by: (a) the individual's attitudes and beliefs towards that behaviour; (b) subjective perception of social norms; and (c) a sense of perceived control (self-efficacy) to perform the behaviour.

Several studies showed that the TPB is a promising theoretical framework to predict sexual risk behaviour in both international (Albarracín, Johnson, Fishbein, & Muellerleile, 2001; Armitage & Conner, 2001; Webb, Joseph, Yardley, & Michie, 2010) and African contexts (Gebreeyesus, Boer, & Kuiper, 2007; Jemmott et al., 2007; Kakoko, Astrom, Lugoe, & Lie, 2006; Schaalma et al., 2009). However, behavioural intentions were found not to be directly related to actual behaviour (Albarracín et al., 2001; Schaalma et al., 2009). Other social and environmental factors were found to moderate the relationship between behavioural intention and actual behaviour (Eaton et al., 2003; Rotheram-Borus, Swendeman, Flannery, et al., 2009; Webb & Sheeran, 2006). To capture the influence of various contextual factors on sexual risk behaviour of young people, the SET (Bronfenbrenner, 1995) is used in this research. According to Bronfenbrenner (1995), behaviour takes place in a dynamic social context where the individual and social context are in constant interaction. Individual behaviour is thus shaped by the social and environmental milieu. The influence of contextual factors such as socioeconomic status, relationship with parents/caregivers, perceived gender norms, and community resources were investigated in this research, based on the research findings of Eaton et al. (2003) and Harrison et al. (2005).

The goal of the research was to investigate current risk behaviour of school-going young people and to explore the underlying reasons for sexual risk behaviour using variables related to the TPB and SET. Another goal was to assess the value of these theories in understanding sexual risk behaviour.

## 2. Methods

A cross-sectional behavioural survey was used. This was part of a baseline study to inform the development of school interventions to reduce sexual risk behaviour of learners.

### 2.1. Sample

A three-stage sampling approach was used. The Department of Basic Education selected 142 schools in specific (mostly rural) districts in four provinces (KwaZulu-Natal, Free State, North West, and Mpumalanga), to participate in the research. These districts were regarded as most adversely affected by the HIV and AIDS epidemic (Shisana et al., 2009) and in need of prevention interventions. The study population thus included learners in the age category 10–18 years who were enrolled in these specific schools. In each school, one class per grade group was selected randomly, by drawing a number from a box. Thirty learners from each selected class were systematically selected from the class list by choosing every second learner on the list, until there were 30 learners from the class. This was done to avoid selection bias. The behavioural survey was completed by 5305 learners. Of these, 1523 were junior learners (Grades 5–9 in primary and middle schools) and 3782 were senior learners (Grade 8–12 in secondary schools).

### 2.2. Survey

A self-report survey assessed sexual risk behaviour of young people and factors identified as potentially underlying risk behaviour (see Table 1). The following questions and scales were included in the survey.

**Table 1.** Assessment scales used.

Scale	Examples of items	Number of items	Response scale	Reliability in this research
Sexual risk scale	I have had sex the past three months I used a condom the last time I had sex	7	Yes/no	0.74 (*n* = 4067)
HIV knowledge	A person can get HIV from having unprotected sex	9	Yes/no	0.59 (*n* = 3763)
Life satisfaction	I have the important things I want in life	5	5-point scale	0.55 (*n* = 3676)
Depression	I feel unhappy, sad or depressed	7	5-point scale	0.68 (*n* = 3737)
Relationship with caregiver	My caregiver supports and encourages me. My caregiver is a role model of how I want to be.	11	5-point scale	0.74 (*n* = 3582)
Personal perception of gender norms	I think a woman should obey her partner	11	5-point scale	0.80 (*n* = 3561)
Perception of community gender norms	People in my community think a woman should obey her partner	9	5-point scale	0.72 (*n* = 3592)
Attitude towards abstinence	Having sex at my age is responsible/ irresponsible	7	Positive/ negative	0.60 (*n* = 2832)
Perceived social norms on abstinence	Most people I respect expect me not to have sex	7	5-point scale	0.70 (*n* = 4067)
Perceived control to abstain	Whether I have sex or not is my own decision	5	5-point scale	0.80 (*n* = 3865)
Attitude towards condom use	Using a condom every time I have sex is acceptable/unacceptable	8	Positive/ negative	0.66 (*n* = 2903)
Perceived social norms on condom use	Most of my friends who are sexually active use condoms	6	5-point scale	0.71 (*n* = 3810)
Perceived control to use condoms	I will be able to use a condom when my friends expect me not to use one	5	5-point scale	0.71 (*n* = 3810)

#### 2.2.1. Demographic information

Questions were asked about gender, age, grade, household composition, and socio-economic status. A socioeconomic status scale was developed where the learners’ perception of their socio-economic status (score 0, 1 or 2) was combined with a figure coupled with the construction of their houses (0 for an informal house (shack) and 1 for a formal house). The socioeconomic status score was expressed on a scale from 0 to 10.

#### 2.2.2. Sexual risk behaviour

Self-reported sexual risk behaviour was assessed using indicators of the United Nations General Assembly Special Session (United Nations General Assembly Special Session on HIV/AIDS [UNGASS], 2009), also used in the Youth Risk Survey (Reddy et al., 2010, 2013) and the household survey (Shisana et al., 2014). The following indicators were used: (a) lifetime sex; (b) current sex (the past three months); (c) forced to have sex; (d) current multiple partners; (e) sex for money/gifts; (f) sex with a person five years and older; (g) sex under the influence of alcohol; (h) having been pregnant or fathered a child; (i) consistent condom use, and (j) condom use at last sex.

For the purpose of analysis, a sexual risk scale was calculated for each senior learner to determine a level of sexual risk. For ethical reasons, junior learners did not complete all sexual risk items. The sexual risk scale consisted of different types of behaviour that could increase a person's risk for HIV. The assumption was that the more types of risk behaviour an individual engaged in, the higher his/her risk exposure. A positive answer to each of the following indicators was given a score of one:

- Lifetime sexual activity.
- Current sexual activity (past three months).
- Current sex with more than one partner (multiple partners).
- Current sex with a partner five years and older (intergenerational sex).
- Currently receiving money or gifts for sex (transactional sex).
- Non-consistent condom use.
- Non-condom use at last sex.

For example, if a learner reported three types of risk behaviour, a score of three was given on a scale of zero to seven. The internal consistency of this scale was 0.74 (*n* = 4067).

#### 2.2.3. HIV-related knowledge

Nine knowledge indicators developed by UNGASS (2009) were used to assess the percentage of learners who could correctly identify ways of HIV transmission and could reject major misconceptions. This scale was a criterion-based measurement to differentiate between learners with high and low knowledge levels; it therefore had a lower alpha coefficient of 0.588 in this research (*n* = 3763).

#### 2.2.4. Psychological well-being

Two existing scales were used to assess psychological well-being. The life satisfaction scale (Diener, Emmons, Larsen, & Griffin, 1985; Pavot & Diener, 2008) was used to evaluate individuals’ subjective satisfaction with their lives. The scale consists of five items rated on a five-point scale. In international research, Diener et al. (1985) obtained high reliability of the scale (test-retest 0.82; alpha coefficient of 0.87). In this research, the reliability was lower (0.55, *n* = 3737).

Depressive mood was assessed through the seven items of the depression scale of Kandel and Davies (1982). Responses were recorded on a five-point scale. Kandel and Davies (1982) reported an alpha coefficient of 0.82, while the reliability in this research was somewhat lower (0.68).

#### 2.2.5. Relationship with parent/caregiver

The learners’ subjective assessment of the quality of their relationship with their caregivers was assessed using 11 items developed for this research from the Safe, Caring, and Child-friendly Schools Framework (Department of Basic Education, 2008). Responses were recorded on a five-point scale. The reliability of the scale was 0.74 for this research.

#### 2.2.6. Perception of gender norms

The learners’ acceptance of traditional gender roles vs. equality in gender roles was assessed using items from the gender relationships scale of Jewkes, Levin, and Penn-Kekana (2002). Learners had to rate the extent to which they agree with statements such as ‘I think a woman should obey her partner’ on a five-point scale. The responses were accumulated into a personal gender norm perception scale with a reliability of 0.80 for this research.

The same questions were reformulated and used to assess individuals’ perceptions of community gender norms. An example of an item from the scale was phrased: ‘People in my community think a woman should obey her partner’. The reliability of the perception of community gender norms scale was 0.72.

#### 2.2.7. Substance abuse

Self-reported use of alcohol, excessive alcohol consumption (more than five drinks per occasion), cigarette smoking, excessive cigarette smoking, and use of illegal drugs the past month were recorded, using yes/no answers. Questions developed for the research of Morojele et al. (2013) were adapted for this research.

#### 2.2.8. Attitudes, social norms, and personal control

Personal attitudes, perceived social norms and personal control related to sexual abstinence and condom use based on the TPB (Ajzen, 1991) were assessed using questions developed for this research.

- *Attitudes*: Descriptive words indicating a positive or negative attitude were provided to learners, requiring them to indicate the statement they agreed with most. For example, ‘Having sex at my age is responsible/irresponsible’. Eight statements were given to assess attitude towards sexual abstinence and condom use. The reliability of the scales was 0.60 and 0.66, respectively.
- *Perceptions of social norms*: Learners’ perceptions of important role players’ (parents, teachers, friends, boy/girlfriend) approval or disapproval of risk behaviour was assessed. This represents the perceived expectations of their behaviour. Seven items were answered on a five-point scale for sexual abstinence and condom use.
- *Personal control (self-efficacy)*: The learners’ confidence to perform protective behaviour in difficult scenarios containing peer group pressure was assessed. Five questions were used in each scale, producing adequate reliability scores.

Two versions of the survey were used. The senior learners (Grades 8–12) completed the whole survey, while specific questions on sexual risk behaviour and condom use were omitted from the survey completed by junior learners (Grades 5–9). This was done because of a request by the provincial Education Departments for ethical reasons.

The questionnaire was piloted in two schools per province that resembled the sample schools to assess learners’ level of understanding and the language used. Adjustments were made accordingly.

### 2.3. Data collection procedures

Officials of the four participating provincial education departments were trained during a two-day training session to conduct the assessment in the selected schools. Training focused on the goal of the assessment, the meaning of each item, how to create a comfortable and confidential situation and how to assure privacy and respect for human rights of each learner. They practised in role play situations.

Data gathering was done using paper and pencil questionnaires in a classroom setting where a Life Orientation educator and the educational official were present. The questionnaire was presented in English. The completion of the questionnaire was mediated question by question. The official translated each question into the vernacular of the learners as they proceeded through the questionnaire to enhance comprehension. To ensure privacy in answers, the questionnaires were completed anonymously. Completed questionnaires were placed in a sealed box to be opened at another venue by the researchers.

### 2.4. Ethical considerations

Ethical clearance was obtained from the Ethics Committee of Humanities at the University of Pretoria, South Africa. The provincial departments of Education and school management teams approved the research. Parents were informed through communiqués from the district offices. Parents of the learners in the sample completed consent forms before data collection took place. Only learners whose parents consented could be included in the sample. Learners included in the sample were informed about the aims of the study and signed assent forms before completing the questionnaires. They were alerted to the fact that participating was completely voluntary. They could decide not to complete the survey and that they need not answer any questions with which they felt uncomfortable. To enhance confidentiality and privacy, no identifying information was asked in the questionnaire. The educational officials were trained to show respect to learners to protect them from harm. The learners did not receive benefits from participation, except some refreshments after completing the survey.

### 2.5. Data analysis

Data was analysed using SPSS version 22. In addition to descriptive data, scale scores were calculated for variables by adding responses to specific items and transposing the result into continuous scales from 0 to 10 (low to high score). Data for senior and junior learners were calculated separately because junior learners did not complete all the questions. Independent t-tests were used to compare condom users and non-condom users to determine variables that influence condom use. To identify variables related to sexual risk of senior learners, biographical data and scale scores were correlated with the sexual risk scale. Variables with significant correlations higher than 0.1 were entered into a stepwise linear regression analysis (Pallant, 2010) to identify the most prominent predictors of sexual risk behaviour.

The sensitive nature of questions related to sexual risk behaviour, resulted in high levels of missing data. To account for missing data and give an accurate presentation of data, percentages are given in terms of the number of respondents who answered each question and not for the sample as a whole. Missing values in sexually related questions hampered the regression analysis. There were 670 learners who left some of the items blank. Based on the nature of the questions, we decided to use the positive answers in the analysis. This implies that we made the assumption that if a learner did not answer a question, this indicated non-agreement with the statement. This allowed for a larger sample size in the regression analysis (*n* = 3637 instead of 2967). To investigate the implications of this decision, a regression analysis with missing values and when missing values were re-coded, were done and resulted in similar findings.

## 3. Results

### 3.1. Demographic description of the learners

Participants were 55.8% female and 44.2% male between 10 and 18 years. The largest group was 16 years and older (48%) and 29% were 13 years and younger. The division of junior and senior learners was made based on the type of school they attended. Junior learners were in primary and middle schools (Grade 5–9), and senior learners were in secondary schools (Grade 8–12). There was thus some overlap regarding grades between the two groups of learners (Table 2). Learners came from North West Province (30.4%), KwaZulu-Natal (25.4%), Mpumalanga (23.7%) and the Free State Province (20.5%).

**Table 2.** Grade groups of junior and senior groups.

	Senior boys (*n* = 1702)	Senior girls (*n* = 2080)	Junior boys (*n* = 624)	Junior girls (*n* = 899)	Total (*n* = 5305)
Grade 5			14.2	12.4	3.7
Grade 6			16.3	13.8	4.2
Grade 7			34.4	32.4	9.3
Grade 8	21.0	18.3	33.5	39.8	24.5
Grade 9	18.7	21.2	1.6	1.6	15.0
Grade 10	33.0	33.8			24.0
Grade11	19.7	17.7			13.3
Grade 12	7.7	8.9			6.1

Note: Values are in percentage.

The majority of learners (64.1%) reported that their families were coping financially with enough money in their households for basic needs (food and clothes). About 10% of the learners indicated that they did not have enough money to meet their basic needs.

Learners were mostly cared for either by both parents (29.6%) or by their mothers as single parents (36.7%). A large group of learners (29.5%) was in the care of grandparents or other family members. About a third of the learners (34.8%) reported that one or both of their parents had died.

### 3.2. Sexual risk behaviour

Lifetime sex was reported by 49.4% of senior boys and 30.5% of senior girls, compared to 16.3% and 5.5% of the junior boys and girls (Table 3). Current sexual activity (during the past three months) was reported by 27.9% of senior boys and 19.4% of senior girls, with fewer juniors currently involved in sexual activities (5.5% boys and 3.6% girls). The average age of sexual debut for sexually active learners (juniors and seniors) was between 15 and 16 years. A third of the learners who reported being sexually active, reported sexual debut before they were 15 years old (43.4% of the sexually active boys, 19.3% of the sexually active girls).

**Table 3.** Reported sexual behaviour.

	Seniors		Juniors		Total (*n* = 5305)
	Boys (*n* = 1702)	Girls (*n* = 2080)	Boys (*n* = 624)	Girls (*n* = 899)	
Had sex in their lifetime	831 (49.4%) (*n* = 1682)	623 (30.5%) (*n* = 2042)	102 (16.3%) (*n* = 624)	48 (5.5%) (*n* = 872)	1623 (30.6%) (*n* = 5304)
Current sex (last 3 months)	408 (27.9%) (*n* = 1462)	299 (19.4%) (*n* = 1541)	29 (5.5%) (*n* = 527)	27 (3.6%) (*n* = 750)	772 (17.8%) (*n* = 4337)
Was forced to have sex	157 (10.6%) (*n* = 1481)	166 (10.0%) (*n* = 1660)	39 (7.1%) (*n* = 549)	36 (4.7%) (*n* = 766)	407 (9.0%) (*n* = 4522)
Had multiple partners past 3 months	271 (31.3%) (*n* = 866)	82 (13.1%) (*n* = 626)			354 (23.4%) (*n* = 1513)
Sex under influence of alcohol	135 (15.7%) (*n* = 860)	58 (9.2%) (*n* = 630)			197 (13.0%) (*n* = 1515)
Sex for money or gifts past 3 months (transactioinal sex)	84 (9.8%) (*n* = 857)	41 (6.5%) (*n* = 631)			127 (8.4%) (*n* = 1512)
Sex with person more than 5 years older (intergenerational sex)	171 (20.0%) (*n* = 855)	142 (22.8%) (*n* = 623)			319 (21.3%) (*n* = 1498)
Have been pregnant or fathered a child	94 (10.9%) (*n* = 862)	132 (20.9%) (*n* = 632)			230 (15.2%) (*n* = 1513)
Condom use every time past 3 months	484 (57.1%) (*n* = 848)	337 (55.3%) (*n* = 609)			831 (56.2%) (*n* = 1479)
Condom used at last sex	590 (70.2%) (*n* = 840)	446 (71.6%) (*n* = 623)			1049 (71.0%) (*n* = 1477)

Notes: Because of missing values, percentages are given in terms of the number of responses to each question. Number of responses are given per question. For ethical reasons, questions about sexual behaviour and condom use were posed only to the senior learners and are reported for senior learners who reported that they have been sexually active.

In this sample, 9% of learners (junior and senior) reported having been forced to have sex, almost equal numbers of boys and girls. More than half of them (55%) told no one about the abuse.

Of the sexually active senior boys, 31.3% reported having multiple sexual partners, 20% intergenerational sex and 15.7% having sex while under the influence of alcohol. Alcohol consumption was more prominent among senior boys than girls: 29% of senior boys in the sample reported current (past 30 days) alcohol use, compared to 16.7% of senior girls. Heavy or binge drinking was reported by 17% of the senior boys and 7.4% of the senior girls.

Of the sexually active senior girls, 20.9% reported having been pregnant, and 22.8% reported intergenerational sex. More than half of the sexually active senior learners (56.2%) reported consistent condom use and more than two-thirds (71%) reported condom use at last sexual encounter.

The sexual risk scale (Table 4) revealed that the majority of senior learners (59.4%) did not report sexual risk behaviours. Among the 40.6% who reported some sexual risk behaviour, 9.2% learners reported engagement in more than three sexually risky behaviours. These learners can be regarded as at extreme risk for HIV. In the sexually active group, 28.5% regarded themselves as at risk for HIV, while 80.5% reported that they know how to protect themselves against HIV.

**Table 4.** Distribution of senior learners on the sexual risk scale.

	Risk score	*N* = 3782	%
No risk	0	2245	59.4
High risk	1	208	5.5
	2	544	14.4
	3	436	11.5
Extreme risk	4	239	6.3
	5	92	2.4
	6	16	0.4
	7	2	0.1

The knowledge indicator showed that learners’ knowledge of HIV was low – 37.8% of all learners in the sample group had accurate HIV knowledge. In an analysis of variance it was found that girls had significantly higher levels of knowledge than boys (*F* = 46.94; *p* < .001). Senior learners also had higher levels of knowledge than junior learners (*F* = 47.11; *p* < .001). Knowledge levels increased with age, ranging from 5.7% of 10-year-olds to 46.1% of 17-year-olds. Further analysis showed that learners had 80% of the answers correct. There were specific areas where respondents lacked knowledge about the transmission of HIV: 37% of the learners indicated that insects could transmit HIV; 31% thought having sex with a healthy-looking person would be safe, while 35% thought birth control pills would protect them from HIV. About 10% of learners believed that HIV could be transmitted through various forms of casual contact.

### 3.3. Factors influencing condom use of senior learners

Senior learners that reported consistent condom use were compared to those that did not report condom use, through independent t-tests (Table 5). Condom users had significantly more knowledge about HIV, they perceived others to support condom use, they had a more positive attitude towards condom use, they reported more perceived self-control with regard to risk behaviour, and they had more positive relationships with their caregivers.

**Table 5.** Variables related to condom use of senior sexually active learners.

Scales	Condom users (*n* = 575)	Non-condom users (*n* = 1039)	*T*
Perceived social norm supporting condom use	6.82	6.27	5.74***
Attitude towards condom use	8.10	7.68	4.14***
HIV knowledge	8.34	8.07	3.61***
Self-control to abstain	6.23	5.78	3.58***
Self-control to use condoms	5.98	5.6	3.35**
Relationship with caregivers	7.39	7.13	3.34**

**p < .01.

***p < .001.

### 3.4. Factors underlying sexual risk behaviour of senior learners

Various factors potentially underlying sexual risk behaviour of young people were assessed in the questionnaire. Variables that correlated significantly and higher than 0.1 with the sexual risk score were entered into a stepwise linear regression analysis with sexual risk as the independent variable. A model was identified that explained 27.4% of the variance of sexual risk (*F*(3368) = 126.658; *p* < .001) (Table 6). The variables related to sexual risk behaviour were the following. Learners’ perceptions of social norms related to abstinence, attitude, and intention to abstain from sex are the variables that affect sexual risk behaviour the most. Furthermore, excessive alcohol consumption and current smoking are related to risk behaviour. Similarly, personal and perceived community gender norms and the quality of the parent-child relationships are social processes that have an important effect on young people's sexual risk behaviour. Age and gender were also prominent factors influencing sexual risk behaviour.

**Table 6.** Regression analysis of variables related to sexual risk.

	Beta	*t* (3631)	*p*-Level
Perceived social norms supporting abstinence	−0.138	−11.067	<.000
Current excessive alcohol use	0.779	10.676	<.000
Intention to abstain	−0.153	−8.882	<.000
Age	0.161	9.393	<.000
Positive attitude towards abstinence	−0.047	−4.680	<.000
Current cigarette smoking	0.389	4.415	<.000
Personal gender norms	0.034	1.975	<.05
Caregiver relationship	−0.067	−4.379	<.001
Gender	−0.149	−3.120	<.01
Perceived community gender norms	0.046	2.778	<.01

The analysis indicates that variables such as life satisfaction, depression, HIV knowledge, and self-control to abstain in the face of peer pressure were, in relation to the other variables, not as prominent predictors of sexual risk behaviour.

The group of learners as a whole (seniors and juniors) experienced an average level of support from significant others to abstain from sex (6.3 on a scale of 0–10). Girls received more support (7.03 on a scale 0–10) than boys (5.5 on a scale 0–10). Of the girls, 71% experienced that their parents/caregivers disapproved of young people becoming sexually active, while 60.3% of their friends also disapproved of being sexually active at a young age. Similarly, boys experienced that 62% of their parents/caregivers disapproved of young people being sexually active. In contrast, boys experience that 71% of their friends encourage them to have sex. Half of the boys (52.6%) in this study (10–18 years old), perceived their friends to be sexually active. Boys thus experience strong peer pressure to be sexually active. Boys also experience less control and self-efficacy related to their sexual decisions (5.0 on a scale of 0–10) than girls (6.04 on a scale of 0–10).

## 4. Discussion

The sexual risk patterns identified in this research compared well with those of recent similar studies among young people in South Africa. The data can, therefore, be seen as an accurate reflection of the risk reported among young people; despite the younger age group included (10–18 years) and that learners came from areas identified as at high risk. In the sample of 5305 learners, 49.4% of the boys and 30.5% of the girls 14 years and older reported having had sex. This compares with the 36% of learners reported being sexually active in the YRBS (Reddy et al., 2013). The rate of early sexual debut remains high when compared to previous research (Pettifor et al., 2009; Shisana et al., 2014). In this study, 33% of sexually active junior and senior learners reported having sex before the age of 15 years – 6.7% of the sample as a whole.

Almost a third of the sexually active senior boys (31.3%) reported having had multiple sexual partners during the past three months. This figure was slightly lower than the 47% reported by Reddy et al. (2013) and slightly higher than the 20.1% reported by Shisana et al. (2014). Even so, it causes concern. Intergenerational sex was prevalent among senior boys as well as girls (20% and 22.8%, respectively), in contrast with Shisana et al. (2014) who found that intergenerational sex is far more prevalent among girls than boys (33.6% vs. 4.1%). Intergenerational sex of boys may be the result of a measurement issue, but needs to be further investigated as it does not receive much attention in the literature. Transactional sex was likely underreported in this study, as 9.8% of boys and 6.5% of girls reported receiving money or goods for sex. It is possible that they associated the question with sex work or were not open about their activities or transactional sex is not as high as anticipated. A high percentage of sexually active girls (20.9%) reported having been pregnant, while 10.9% boys acknowledged that they fathered a child. These results are similar to the findings of the YRBS (Reddy et al., 2013). Consistent condom use among senior sexually active learners (56.2%), and condom use at last sexual encounter (71%) was higher than previously reported (Reddy et al., 2013; Shisana et al., 2014), but still cause for concern. Learners’ perceptions of their own risk of exposure to HIV was low. In the sexually active group, 28.5% regarded themselves as at risk of HIV. The low perception of personal risk is echoed in the UNAIDS (2014a) report where 82% of South African young people are of the opinion that they are not at risk of HIV infection.

Although 87.5% of learners reported that they had learned about HIV in their schools, there seems to be a lack of HIV knowledge. Only 37.8% of the sample as a whole had accurate knowledge about HIV. It was found that knowledge levels increased with age. While only 5.7% of 10-year-olds had accurate knowledge, 46.1% of the 17-year-olds answered all questions correctly. Shisana et al. (2014) found even lower levels of HIV knowledge among young people aged 15–24 years (28.6%). Prevention interventions, therefore, have to focus on increasing HIV knowledge, specifically the areas identified as problem areas, as the core of school interventions.

Inconsistencies in data from various studies highlighted the sensitivity of the topic and the influence of the choice of respondents and the data collection process on the research findings. Self-report of sensitive behaviour may not always be accurate and can be influenced by many internal and external factors. There is not a way to accurately verify the data (Catania, Gibson, Chitwood, & Coates, 1990). From the findings of various studies, it is possible to identify common behavioural trends of young people that can be used in planning prevention efforts.

This research shows that despite two decades of HIV education in schools (Department of Education, 1998, 2003, 2011), the consistently high sexual risk behaviour of learners and the lack of HIV knowledge emphasise the lack of effective interventions and the importance of relooking HIV prevention in schools. There is no shortage of research, programmes, and guidelines for HIV prevention in schools (Anderson, Panchaud, Singh, & Watson, 2013; Biddlecom, Hessburg, Singh, Bankole, & Darabi, 2007; Department of Basic Education, 2003, 2011; Department of Health, 2003; Family Health International, 2009; Klepp, Flisher, & Kaaya, 2006; Ross, Dick, & Ferguson, 2006; Senderowitz & Kirby, 2006). This research demonstrates that a gap still exists between existing knowledge about HIV prevention and interventions implemented in South African schools. HIV interventions are often short-term and knowledge-driven, and many challenges exist in the implementation of HIV prevention in schools (Aggleton, Yankah, & Crewe, 2011; Frempong, Reddy, & Kanjee, 2011; Shefer & MacLeod, 2015; Visser, 2005).

### 4.1. Factors predicting sexual risk behaviour

This research identified several variables underlying sexual risk behaviour that should be targeted for prevention interventions to be effective. Perceived social norms played the most important role in encouraging sexual risk behaviour. Perceived social norms refer to the behaviour young people observe amongst their peers; what they perceive others approve of and expect of them (Ajzen, 1991). Peer group pressure is thus the strongest predictor of risk behaviour, particularly for males. Sexual risk behaviour was also prominent among learners that do not intend to abstain from sex or have negative attitudes towards abstinence. These results show that variables from the TPB (intentions, attitude, and perceived social norms) were strong predictors of sexual risk behaviour. Unlike previous research that highlighted perceived behavioural control as the most prominent explanation of intention to implement protective behaviour (Albarracín et al., 2001; Armitage & Conner, 2001; Jemmott et al., 2007; Kakoko et al., 2006; Schaalma et al., 2009), perceived social norms were the strongest predictor of risk behaviour in the present study. Learners’ perceived behavioural control or self-efficacy was fairly low in this study. Boys, in particular, did not experience having enough control to make decisions about their behaviour in the face of difficult situations involving peer group pressure.

Similarly, this research confirms the value of the TPB in predicting condom use. The learners that reported consistent condom use differed from non-condom users in their attitude towards condom use, perceived social norms, and feeling in control of sexual decisions and condom use. This confirms the results of previous research (Hendriksen, Pettifor, Lee, Coates, & Rees, 2007; Schaalma et al., 2009) that the TPB is of value in predicting condom use. The TPB thus has potential as a framework to explain sexual risk behaviour and to plan preventive interventions.

HIV risk behaviour was prominent for older learners and males. More males than females reported being sexually active, having multiple partners, and having sex under the influence of alcohol. Males also reported experiencing high levels of peer pressure to engage in sexual risk behaviour. In contrast to the current prevention strategies that focus largely on young females (Shisana et al., 2014; UNAIDS, 2014b), this research demonstrated the importance of prevention interventions amongst young males. It is during the adolescent years that young men develop their behavioural patterns and beliefs about gender norms. If interventions can address the peer group and gender norms young men are exposed to, a difference can be made in their current and future high-risk behaviour and can furthermore impact risk situations for young females.

In addition to individual variables, social and cultural factors such as peer group and community gender norms and a culture that promotes substance abuse, influenced learners’ sexual risk behaviour. These are social processes not under the control of the individual, as explained by the SET (Bronfenbrenner, 1995). Risk is embedded in broader social structures (Rotheram-Borus, Swendeman, Flannery, et al., 2009) that need to be changed to shape healthier behaviour.

Learners that ascribed to traditional gender norms or perceived such norms in their community, engaged in more sexual risk behaviours. These young people attach more status to males than females, believe that males can have more than one girlfriend, and that a woman does not have the right to refuse sex with her partner (Jewkes et al., 2002, 2010). These beliefs encourage risk behaviour amongst males, such as having multiple partners, and disempower women to protect themselves from HIV and take control of their sexual health. The development and reaction towards gender norms should, therefore, form part of HIV prevention strategies for young people.

Excessive alcohol consumption and cigarette smoking predicted sexual risk behaviour. This confirms previous research (Taylor, Dlamini, Kagoro, Jinabhai, & De Vries, 2003; Woolf-King & Maisto, 2011). The relationship between substance use and sexual risk was explained by Reddy et al. (2010), who found that different risk behaviours cluster together, especially for male respondents. They found that some individuals who have risk-prone profiles, engage in more than one type of risky behaviour. If one wishes to address HIV risk, various forms of substance abuse therefore also need to be addressed.

A positive caregiver relationship was identified as a protective factor against sexual risk behaviour. The quality of the relationship with a parent or caregiver has been consistently identified as an important factor in the mental health and healthy behaviour choices of HIV-affected adolescents in South Africa (Cluver, Boyes, Orkin, & Sherr, 2013; Cluver, Orkin, Boyes, Sherr, Makasi, et al., 2013; Knerr, Gardner, & Cluver, 2013). Despite these findings, parents or caregivers have not been involved in HIV prevention efforts in schools.

### 4.2. Factors not predicting sexual risk behaviour

Four factors that were expected to underlie sexual risk behaviour, but were not prominent in the regression analysis, need to be highlighted. *Socio-economic status* did not predict sexual risk behaviour, in contrast with previous research (Cluver, Boyes, Orkin, Pantelic, et al., 2013; Cluver, Boyes, Orkin, & Sherr, 2013; Cluver, Orkin, Boyes, Sherr, Makasi, et al., 2013; Leclerc-Madlala, 2008; Wight et al., 2006). The current research included at-risk schools, mostly from low socio-economic communities. This limited the variance in the socio-economic variable. Additionally, a very crude measure of socio-economic status was used, depending on the learners’ subjective assessment of having enough or not enough money for basic needs. The finding in this research does *not* have implications for the value of poverty alleviation interventions as HIV prevention strategy.

*HIV knowledge* was the second factor that did not predict sexual risk behaviour. In this study, 37.8% of the learners had accurate HIV knowledge. A comparison of plots of the two variables (HIV knowledge and sexual risk) showed that the two variables had different structures that were unrelated. Unlike the results for sexual risk behaviour, HIV knowledge differentiated condom users from non-condom users. Learners thus need accurate knowledge in their decision to use condoms. This research consequently demonstrated that various other factors had a stronger influence on sexual risk behaviour than the level of HIV knowledge. Prevention packages should therefore not only focus on HIV knowledge, but should address the other variables underlying sexual risk behaviour.

*Perceived control* of sexual behaviour (self-efficacy) did not predict sexual risk behaviour as expected (Schaalma et al., 2009). Perceived control focused on learners’ preparedness for own decision-making and their skills to resist peer pressure. The learners’ sense of control was rather low (5.7 on a scale of 0–10). Learners, especially boys, felt influenced by the social norms and peer pressure and did not perceive that they have enough control to make their own decisions. On the other hand, learners’ sense of personal control distinguished condom users from non-condom users.

*Mental health*, as assessed by life satisfaction and depression, also did not play a role in learners’ sexual risk behaviour as expected. It seems that the social processes influenced behaviour more than individual factors.

These results demonstrate that interventions have to focus on both individual and social levels to make a difference in young people's risk behaviour. The broader structural factors that shape and constrain individual behaviour (Gupta et al., 2008) need to be addressed to enable individual change.

## 5. Recommendations

The results of the research revealed that 40.6% of the learners reported sexual risk behaviour on the sexual risk scale. Risk behaviour differs between age and gender groups. HIV prevention therefore has to be targeted at the needs of specific groups. Primary prevention and health promotion should be the focus for learners who are not likely at risk, so as to encourage delay of sexual debut and knowledge about condom use (mostly at primary school). Risk reduction programmes will focus on the learners who are at risk of HIV to promote condom use and decrease high-risk sexual behaviour. This research emphasised that HIV prevention should focus on individual and structural factors to change community gender norms and relationships between young people. Based on the results of this research, HIV prevention in schools should include the following:

*Accurate information* about HIV risk and protective behaviour should form part of training in sexual and reproductive health, which should be the core of a school intervention. Knowledge needs to be conveyed in a way that learners can make it part of their own lifestyle, such as using drama interventions (Gausset, 2001). Most current HIV prevention programmes in schools focus solely on HIV knowledge (Department of Education, 2011).

In addition to awareness and knowledge, HIV prevention should include a *behaviour change component* to assist young people in adopting a healthy lifestyle. The TPB showed promise as a theoretical framework to promote a healthy lifestyle which includes delayed sexual debut, reduced number of partners (especially intergenerational partners), protected sex and knowledge of HIV status. Positive attitudes towards abstinence and condom use as protection against HIV should be highlighted. Most of all, learners should be challenged to develop a critical awareness (Freire, 1993) of the influence that peer group and community gender norms have on their behaviour. This involves an intellectual understanding of how current ways of thinking about gender and sexuality negatively influence their decision making. It also involves dialogue and critical discussion between boys and girls to negotiate new ways of relating that is healthy for both parties and can lead to gender transformation and gender justice (Shefer & MacLeod, 2015). Especially boys need to challenge gender norms that expect of them to be sexually active, have many partners and use substances to prove their masculinity (Dworkin, Hatcher, Colvin, & Peacock, 2013; Lynch, Brouard, & Visser, 2010; Mfecane, 2010). Such honest conversations between young people, facilitated by a trained adult, have shown to make a difference in how young people make decisions and relate to each other.

Young people also need to develop confidence in their ability to take control of their own sexual decision making. They should have the skills to resist peer pressure and other contradicting influences. Positive peer group pressure can be strengthened through leadership development as an alternative means of influencing young people's behaviour.

These interpersonal negotiations should be duplicated on broader community levels, including parents and caregivers so that adults can discuss and model positive gender relationships for the youth. Social transformation is needed, focusing on economic and social empowerment of women to reduce intergenerational sexual relationships and gender-based violence (Hendriksen et al., 2007; Stirling et al., 2008). Large volumes of research and advocacy documents exist to promote equality in gender roles and empowerment of women to prevent HIV infection (e.g. Jewkes et al., 2010; Mantell et al., 2006; Stirling et al., 2008; Strebel et al., 2006). These principles need to be implemented as part of school interventions and on the community level to model positive gender relationships. Change in community norms and social structures is necessary to sustain behaviour change structures (Rotheram-Borus, Swendeman, Flannery, et al., 2009).

This research provides evidence that the quality of young people's relationships with their parents or caregivers is a protective factor against sexual risk behaviour. Parents/caregivers should, therefore, be involved in school-based HIV prevention efforts, especially to learn parenting skills and how to communicate about sensitive topics. Training in parenting skills could improve caregivers’ understandings of child development and to improve the quality of parent-child relationships (Knerr et al., 2013). Additionally, parents/caregivers are role models and the primary sources of information and guidance for young people related to sexual development (Akers, Holland, & Bost, 2011; Goodnight et al., 2014). Previous research showed that young people exposed to communication about sex at home showed higher self-efficacy, positive attitudes and intentions, responsible decision making, limited risky sexual behaviour, and increased knowledge about sex (Bastien, Kajula, & Muhwezi, 2011; Coetzee et al., 2014). Effective caregiver-youth communication about sex can, therefore, protect young people from a range of risk behaviours (Bastien et al., 2011; Goodnight et al., 2014; Rotheram-Borus, Flannery, Rice, & Lester, 2005). Parents/caregivers should thus be empowered to overcome the barriers (De Palma & Francis, 2014; Moletsane, 2011) and develop the skills (Bastien et al., 2011; Goodnight et al., 2014; Phetla et al., 2008) to effectively talk to young people about sensitive issues like sex, pregnancy, contraceptives, and HIV prevention. There is evidence of effective interventions for caregivers to provide sexual education for young people (Akers et al., 2011).

## 6. Limitations

The sample of learners was not a random sample representative of the population of young people in the country. The study was conducted in four provinces in schools selected because of a potentially high risk for HIV and lack of interventions in the school. Bias can exist based on the location of the schools and the socioeconomic climate in the community, as well as the unique characteristics of the schools involved. Additionally, learners had various degrees of exposure to prevention programmes that may have influenced their knowledge, attitudes, and behaviour. Despite the fact that the results of this study cannot be generalised to the population as a whole, the large sample size allows for identification of general behaviour patterns.

Language used could have played a role in the accuracy of data. The questionnaire was presented in English and translated where necessary during completion. Some misunderstandings could have occurred. Questions were formulated based on the concepts of international HIV literature, which may not have been part of the conceptual framework of 10–15-year-olds in a rural area. This might have contributed to inconsistencies and missing responses.

Earlier research identified many constraints in the accuracy of self-reported sexual behaviour (Catania et al., 1990). The social and personal desirability of responses can influence the data, especially since there are significant differences between genders in this regard. Several patterns of over- and underreporting were found to interact in collecting data on sexual behaviour. Girls were found to often under-report sexual activity (Wislar & Fendrich, 2000) while boys, in line with the peer norms they perceive, may conversely over-report (Catania et al., 1990). Learners may have difficulty conveying sensitive information despite all assurances given by data collectors. The results on self-reported sexual behaviour patterns thus have to be interpreted against the complexities involved in gathering sensitive data, but the current research is not unique in this regard. It applies to all similar research.

## 7. Conclusions

The research confirmed many previous research findings about sexual risk behaviour among young people that can contribute to identifying common patterns. The research also provided evidence of underlying factors of sexual risk that was often found in qualitative research. The research highlighted the importance of re-thinking school-based HIV prevention, because of the high levels of sexual risk behaviour that still exist after two decades of school-based interventions. The value of the TPB to understand and influence sexual risk behaviour was demonstrated. Of similar importance, the research identified peer group and community gender norms, a culture of substance abuse and quality of relationship with caregivers as underlying young people's sexual risk behaviour. HIV prevention should therefore not focus on young people's knowledge, skills, and behaviour alone. It is even more important to raise critical awareness of the influence of gender and peer group norms on sexual risk behaviour. These broader structural factors shape and constrain individual behaviour (Gupta et al., 2008). Awareness of these factors can empower young people to re-negotiate and transform their own healthier social norms. The protective power of positive parent-child relationships should also be explored and enhanced. The research also focused on the importance of interventions for boys/men to renegotiate gender norms that will allow them to make healthy choices.

Sustainable change requires shifting community and peer group norms and the broader social structures that shape behaviour underlying HIV-epidemics and the social problems that accompany it. This research emphasised the statement of Stirling et al. (2008): ‘Nothing less than social transformation is needed now to turn this epidemic around’ (p. S3).
